# Temporal trends in colorectal cancer mortality rates (1999–2022) in the United States

**DOI:** 10.1002/cnr2.2012

**Published:** 2024-03-05

**Authors:** Fariha Ilyas, Eiman Ahmed, Hassam Ali, Maheen Ilyas, Shiza Sarfraz, Mahnoor Khalid, Muhammad Khalaf, Prashant Reddy Mudireddy

**Affiliations:** ^1^ Department of Internal Medicine ECU Health Medical Center Greenville North Carolina USA; ^2^ Department of Library and Information Science Rutgers University New Brunswick New Jersey USA; ^3^ Department of Gastroenterology ECU Health Medical Center Greenville North Carolina USA; ^4^ Osteopathic Medical Student III, New York Institute of Technology College of Osteopathic Medicine Old Westbury New York USA; ^5^ Department of Internal Medicine Quaid‐e‐Azam Medical College Bahawalpur Punjab Pakistan; ^6^ Department of Internal Medicine Foundation University Medical College Rawalpindi Pakistan

**Keywords:** colorectal neoplasms, disease progression, epidemiology, mortality, population surveillance

## Abstract

Colorectal cancer (CRC) ranks as the third leading cause of cancer‐related deaths in the United States (U.S.). Our study aims to analyze CRC mortality patterns in the U.S., focusing on gender and age groups from 1999 to 2022. We analyzed Age‐Adjusted Mortality Rates (AAMRs) for CRC‐related deaths using the CDC Wide‐ranging Online Data for Epidemiologic Research (CDC WONDER) database and assessed differences between age and sex. CRC‐related mortality decreased significantly from 1999 to 2011 (−2.81% APC) and from 2011 to 2020 (−1.95% APC) but a not significant uptrend from 2020 to 2022 (2% APC). Males experienced a more significant decrease. Among age groups, crude mortality decreased until 2020, except in age group 45–54, which showed an annual increase in mortality of 0.9% from 2004 to 2022. Furthermore, individuals aged 75–84 and 85+ saw a nonsignificant annual increase of 1.8% and 4.5% from 2020 to 2022, respectively. Our study highlights a significant decline in age and gender‐specific CRC‐related mortality from 1999 to 2020. However, the worrisome uptrend observed in the younger age group of 45–54 emphasizes the importance of implementing targeted public health measures and evidence‐based interventions.

## 1 INTRODUCTION

Colorectal cancer (CRC) emerges as a significant global public health challenge, projected to surge by 60% with over 2.2 million new cases and 1.1 million deaths anticipated by 2030.^1^, ^2^, ^3^ The prevalence of CRC burden exhibits substantial variability, with over two‐thirds of total cases and approximately 60% of all related deaths concentrated in nations characterized by a high or very high human development index.^3^ Various environmental and genetic factors, including lifestyle, dietary habits, family history, and metabolic health conditions, contribute to CRC risk.^4^ Despite progress in early detection and therapeutic measures, colorectal cancer (CRC) persists as the third most common cause of cancer‐related deaths in the United States.^1^ It is imperative to monitor CRC mortality trends to evaluate existing interventions effectively and pinpoint areas for improvement.^1^, ^2^ Moreover, the recent surge in early‐onset colorectal cancer, coupled with the subsequent revision of the recommended screening protocol by the US Preventive Service Task Force (USPSTF), emphasizes the crucial role of routine screening for individuals aged 45–75 years.^2^ This study aims to scrutinize and discuss trends in CRC‐related mortality rates among individuals aged 35 and above in the United States from 1999 to 2022. The nuanced analysis of mortality trends offers valuable insights into the overall impact of preventive methods, such as screening protocols and advancements in treatment, allowing us to gauge the effectiveness of the multifaceted strategies employed in addressing this significant public health challenge. By conducting this analysis, the research aims to provide valuable insights into the effectiveness of current strategies, identify potential avenues for enhancing interventions, and ultimately inform public health initiatives geared toward reducing the burden of colorectal cancer and improving patient outcomes.

## 2 METHODS

### 2.1 Study population

Data obtained from the Centers for Disease Control and Prevention (CDC), were used to analyze colorectal cancer related mortality rates per 100 000 population from 1999 through 2022. CRC patients were identified using ICD‐10 diagnostic codes C18–C19. Additional demographic information is not provided in CDC wonder database to protect patient privacy.^3^

All patients with ages 35 years or above were included in our study.^3^ We excluded patients under the age of 35 due to their lower mortality rates, as including them could introduce confounding factors and because they did not fall within our population of interest.

### 2.2 Statistical analysis

Age‐adjusted mortality rates (AAMRs) were utilized for the study and standardized to the 2000 U.S. population. For age subgroups, crude mortality rates (CMR) were analyzed. Trends were established using the Joinpoint Trend Analysis Software (version 5.0.1), which assesses temporal trends in the Average Percentage Change (APC). The analysis with Joinpoint involved a Monte Carlo permutation test and a *t*‐test to accurately detect “joinpoints”—the points at which there is a statistically significant change in the trend.^4^ This combination allows for the detection of both gradual and abrupt trend changes over time. Additionally, we included log‐linear regression models in our analysis to provide a more comprehensive understanding of the trends in age‐standardized mortality rates. This regression modeling approach was vital for understanding the direction and strength of these trends.^4^ Trends were regarded as increasing or decreasing when the APC significantly differed from 0 (*p* < .05) using a two‐sided test.

## 3 RESULTS

**FIGURE 1:**
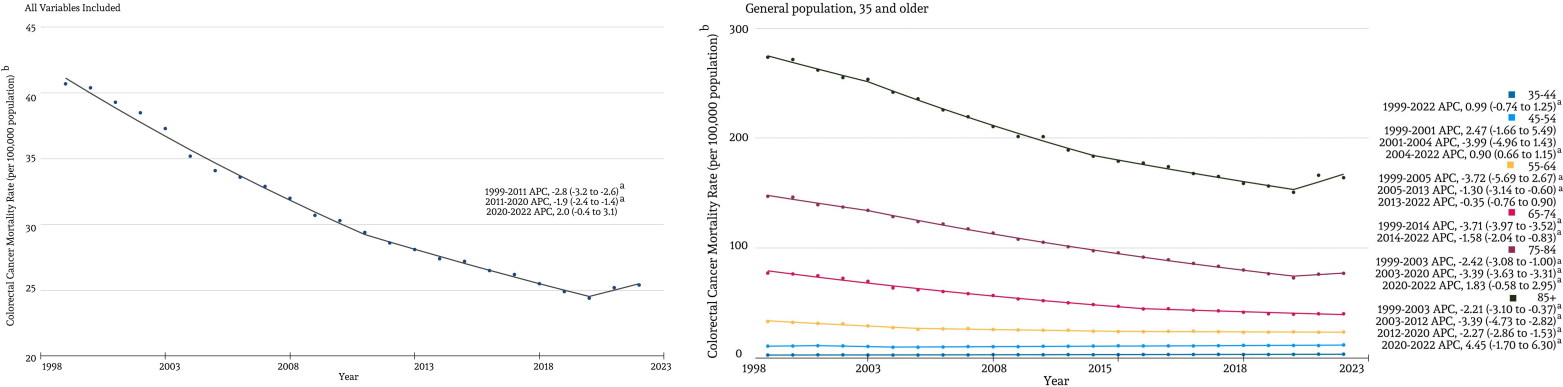
Trends in colorectal cancer mortality from 1999 to 2022 in the United States, stratified by age groups. Total rates are reported as age adjusted mortality rate, age groups are reported as crude mortality rates.

During 1999–2022, 1 270 442 people died from CRC. Subjects were 52% male. CRC mortality rates per 100 000 population declined from 40.7 in 1999 to 25.4 in 2022, with an APC for the study period (1999–2022) −2.23% (95% CI, −2.45 to −2.02%). The APC was −2.81% (95% CI, −3.20% to −2.59%) from 1999 to 2011, −1.90% (95% CI, −2.36% to −1.39%) from 2011 to 2020 and 1.95% (95% CI, −0.42% to 3.09%) from 2020 to 2022 for overall population (Figure 1).

**FIGURE 2:**
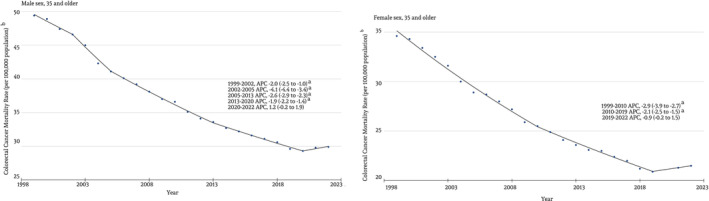
Trends in Colorectal Cancer Mortality from 1999 to 2022 in the United States, stratified by gender.

Among males, there was a decrease from 49.4 in 1999 to 29.9 in 2022, with an APC of −2.40% (95% CI, −2.61% to −2.19%). Three joinpoints were identified with a significant decline, as shown in Figure 2. Among females, there was a decrease from 34.6 in 1999 to 21.5 in 2022, with an APC of −2.30% (95% CI, −2.48% to −2.09%). Two joinpoints were identified, with a significant decline, as shown in Figure 2.

In age‐stratified analyses, crude mortality trends have been steadily decreasing for all age groups until 2020, except in age group 45–54, which showed an increase in mortality by 0.9% (95% CI, 0.66%–1.15%) annually from 2004 to 2020 (Figure 1). All additional age subgroups showed a significant decline in mortality for the study period, with variable joint points identified, as shown in Figure 1.

## 4 DISCUSSION

Our examination of colorectal cancer (CRC)‐related mortality trends from 1999 to 2022 revealed a substantial decline in mortality rates from 1999 to 2022, but a nonsignificant uptick from 2020 to 2022. The decrease until 2020 is attributed to significant advancements in early detection and prevention, instrumental in reducing CRC incidence and mortality rates.^5^ Recognizing the importance of organized screening programs, we advocate for diverse screening methods such as sigmoidoscopy, guaiac‐based fecal occult‐blood testing (FOBT), colonoscopy, and fecal immunochemical tests (FIT).^6^, ^7^ We propose an inclusive strategy by emphasizing integrating fecal testing alongside colonoscopies to enhance screening rates and accommodate patient preferences.^8^

Unraveling the pathophysiology of CRC has significantly contributed to the decline in mortality rates, offering diverse treatment options, including endoscopic and surgical excision, immunotherapy, radiotherapy, targeted therapy, and minimally invasive procedures.^9^ Although effective, these treatments have notably doubled overall survival for advanced disease to 3 years, while individuals with nonmetastasized disease continue to experience favorable outcomes.

However, the significant increase in mortality in the age group of 45–54 from 2004 to 2022 may be linked to rising incidence rates, especially in early‐onset CRC.^10^ Recent recommendations for screening starting at age 45 acknowledge this trend, but its impact on disease burden remains uncertain. Our findings emphasize the need for interventions to enhance screening participation in the 45–54 age group, considering the surge in CRC incidence and low on‐time screening rates at age 50.^11^ The rise in stool testing adoption among underserved communities is seen as a potential solution to improve screening rates, particularly during healthcare disruptions like the COVID‐19 pandemic. The birth cohort effect since the 1950s may also contribute to the observed mortality increase, emphasizing the urgency of identifying factors and implementing targeted interventions for this age group.

Our study also unveiled a nonsignificant increase in mortality rates from 2020 to 2022, impacting both genders disparately and notably affecting individuals aged 75–84 and those aged 85 and older. This surge could be partly attributed to the national emergency declaration in response to the COVID‐19 pandemic, resulting in an 80%–85% decline in CRC screening volume.^12^, ^13^ Timely screening, essential for improved prognosis and reduced cases and deaths, faced challenges during the pandemic. A study by Worthington et al. underscored the global impact of COVID‐19 disruptions on organized colorectal cancer screening, indicating potential additional CRC cases and deaths globally from 2020 to 2050.^14^

Elderly patients, constituting one‐third of new CRC diagnoses globally, face unique challenges with inadequate treatment, contributing to poor adherence and suboptimal treatment quality.^15^ Time‐to‐treatment initiation delays during the pandemic significantly elevated mortality risk.^14^ The unexpected rise in mortality during the pandemic emphasizes the critical need for ongoing monitoring and targeted health interventions, particularly for older populations.

Our study's strengths include its broad scope and diverse data sources, enhancing its potential generalizability. Spanning 23 years and using the CDC's online epidemiologic database, it offers a thorough analysis of colorectal cancer (CRC) mortality trends, including during the COVID‐19 peak. This period, from 1999 to 2022, highlights the impact of COVID‐related screening declines, underlining the need for timely CRC screening.

However, the study has limitations. Relying on ICD codes and death certificates may lead to underrepresented CRC mortality. Inaccuracies in reporting, especially regarding race and ethnicity, could skew results and hinder our ability to address mortality disparities. The inclusion of the COVID‐19 period adds complexity. These factors suggest caution in interpreting our findings and call for further research to improve CRC data accuracy.

## AUTHOR CONTRIBUTIONS

**Fariha Ilyas:** Conceptualization (equal); data curation (equal); writing – original draft (equal); writing – review and editing (equal). **Eiman Ahmed:** Supervision (equal); validation (equal); visualization (equal). **Hassam Ali:** Conceptualization (equal); data curation (equal); formal analysis (equal); funding acquisition (equal); project administration (equal). **Maheen Ilyas:** Software (equal); supervision (equal); validation (equal); writing – original draft (equal); writing – review and editing (equal). **Shiza Sarfraz:** Methodology (equal); project administration (equal); writing – original draft (equal); writing – review and editing (equal). **Mahnoor Khalid:** Data curation (equal); formal analysis (equal); resources (equal). **Muhammad Khalaf:** Software (equal); writing – original draft (equal); writing – review and editing (equal). **Prashant Reddy Mudireddy:** Supervision (equal); writing – original draft (equal); writing – review and editing (equal).

## CONFLICT OF INTEREST STATEMENT

All the authors declare that there is no conflict of interest regarding the publication of this research study. They have no financial, personal, or professional affiliations that could be perceived as influencing the objectivity, integrity, or impartiality of the research or its outcomes.

## ETHICS STATEMENT

This study is exempt from Institutional Review Board (IRB) review as it utilizes publicly available, de‐identified data sourced from the CDC WONDER database and is retrospective in nature.

## Data Availability

Data sharing is not applicable to this article as no new data were created or analyzed in this study.

## References

[1] Siegel RL , Wagle NS , Cercek A , Smith RA , Jemal A . Colorectal cancer statistics, 2023. CA A Cancer J Clin. 2023;73(3):233‐254. doi:10.3322/caac.21772 36856579

[2] Gupta S . Screening for colorectal cancer. Hematol Oncol Clin N Am. 2022;36(3):393‐414. doi:10.1016/j.hoc.2022.02.001 PMC9167799 35501176

[3] CDC WONDER. https://wonder.cdc.gov/. Accessed May 30, 2023

[4] Kim HJ , Fay MP , Feuer EJ , Midthune DN . Permutation tests for joinpoint regression with applications to cancer rates. Stat Med. 2000;19(3):335‐351. doi:10.1002/(sici)1097-0258(20000215)19:3<335::aid-sim336>3.0.co;2-z 10649300

[5] Kahi CJ , Imperiale TF , Juliar BE , Rex DK . Effect of screening colonoscopy on colorectal cancer incidence and mortality. Clin Gastroenterol Hepatol. 2009;7(7):770‐775; quiz 711. doi:10.1016/j.cgh.2008.12.030 19268269

[6] Zorzi M , Fedeli U , Schievano E , et al. Impact on colorectal cancer mortality of screening programmes based on the faecal immunochemical test. Gut. 2015;64(5):784‐790. doi:10.1136/gutjnl-2014-307508 25179811

[7] Schoen RE , Pinsky PF , Weissfeld JL , et al. Colorectal‐cancer incidence and mortality with screening flexible sigmoidoscopy. N Engl J Med. 2012;366(25):2345‐2357. doi:10.1056/NEJMoa1114635 22612596 PMC3641846

[8] Qaseem A , Harrod CS , Crandall CJ , et al. Screening for colorectal cancer in asymptomatic average‐risk adults: a guidance Statement from the American College of Physicians (version 2). Ann Intern Med. 2023;176(8):1092‐1100. doi:10.7326/M23-0779 37523709

[9] Dekker E , Tanis PJ , Vleugels JLA , Kasi PM , Wallace MB . Colorectal cancer. Lancet. 2019;394(10207):1467‐1480. doi:10.1016/S0140-6736(19)32319-0 31631858

[10] Stoffel EM , Murphy CC . Epidemiology and mechanisms of the increasing incidence of colon and Rectal cancers in young adults. Gastroenterology. 2020;158(2):341‐353. doi:10.1053/j.gastro.2019.07.055 31394082 PMC6957715

[11] Dougherty MK , Brenner AT , Crockett SD , et al. Evaluation of interventions intended to increase colorectal cancer screening rates in the United States: a systematic review and meta‐analysis. JAMA Intern Med. 2018;178(12):1645‐1658. doi:10.1001/jamainternmed.2018.4637 30326005 PMC6583619

[12] Chen RC , Haynes K , Du S , Barron J , Katz AJ . Association of cancer screening deficit in the United States with the COVID‐19 pandemic. JAMA Oncol. 2021;7(6):878‐884. doi:10.1001/jamaoncol.2021.0884 33914015 PMC8085759

[13] Carethers JM . Insights into disparities observed with COVID‐19. J Intern Med. 2021;289(4):463‐473. doi:10.1111/joim.13199 33164230 PMC9325576

[14] Wells CR , Galvani AP . Impact of the COVID‐19 pandemic on cancer incidence and mortality. Lancet Public Health. 2022;7(6):e490‐e491. doi:10.1016/S2468-2667(22)00111-6 35660207 PMC9159732

[15] Yamano T , Yamauchi S , Kimura K , et al. Influence of age and comorbidity on prognosis and application of adjuvant chemotherapy in elderly Japanese patients with colorectal cancer: a retrospective multicentre study. Eur J Cancer. 2017;81:90‐101. doi:10.1016/j.ejca.2017.05.024 28622612

